# Digital Maturity and Its Measurement of General Practitioners: A Scoping Review

**DOI:** 10.3390/ijerph20054377

**Published:** 2023-02-28

**Authors:** Timo Neunaber, Sven Meister

**Affiliations:** 1 Health Informatics, Faculty of Health/School of Medicine, Witten/Herdecke University, 58455 Witten, Germany; 2 Department Healthcare, Fraunhofer Institute for Software and Systems Engineering, 44227 Dortmund, Germany

**Keywords:** digital maturity, primary care, general practitioners, digitalization

## Abstract

The work of general practitioners (GPs) is increasingly characterized by digitalization. Their progress in digitalization can be described by the concept of digital maturity and measured using maturity models. The aim of this scoping review is to provide an overview of the state of research on digital maturity and its measurement in primary care, specifically for GPs. The scoping review was conducted according to Arksey and O’Malley, considering the reporting scheme for PRISMA-ScR. For the literature search, we used PubMed and Google Scholar as the main sources of information. A total of 24 international, mostly Anglo-American studies, were identified. The understanding of digital maturity varied widely. In most studies, it was understood in a highly technical way and associated with the adoption of electronic medical records. More recent, but mostly unpublished, studies have attempted to capture overall digital maturity. So far, the understanding of digital maturity of GPs is still very diffuse—the research literature is still in its infancy. Future research should therefore aim to explore the dimensions of digital maturity of GPs to be able to develop a consistent and validated model for measuring digital maturity.

## 1. Introduction

The potential applications of digitalization in general practices and primary care organizations are vast [1]. Examples of use range from the simplification of administrative processes and online appointment scheduling to the direct use of telemedicine applications in the treatment of patients [2,3]. Despite the wide-ranging potential of digitalization, international comparisons show that the spread and use of digital applications in primary care varies widely [4,5]. The World Health Organization identifies an assessment of digital maturity of a health system as a key to promoting the development and use of digital technologies, based on which national investments can be made [6].

### 1.1. General Practitioners in the Context of Digital Transformation

General practitioners (GPs) are the most important actors in primary care in European countries [7]. According to the Declaration of Alma, primary care ensures the first contact of individuals, families, and communities with the health system [8]. The World Organization of Family Doctors (WONCA) defines GPs as trained physicians who are “primarily responsible for the provision of comprehensive and continuing care to every individual seeking medical care irrespective of age, sex and illness” [9] (p. 12). In their professional role, they undertake tasks ranging from disease prevention and treatment to the promotion of patient self-management [8,9]. In this context, GPs operate mainly in individual and group practices [5]. In terms of referral to specialists, primary care varies across Europe. While in countries such as the Netherlands or Finland, GPs take on the role of gatekeepers and consultation with specialists is not possible without referral, there are no restrictions in countries such as Austria and Greece [7]. 

Working in general practices is increasingly characterized using digitalization. Digitalization is understood as digital transformation in the context of this study. Ebert and Duarte describe it with the adoption of “disruptive technologies to increase productivity, value creation, and the social welfare” [10] (p. 1). Digital transformation is also characterized by, among other things, inevitability, rapidity, and uncertainty [11]. The reasons for increasing digitalization in a general practice are manifold. Different areas and business processes of a general practice can be affected by digitalization. Before visiting the practice, a digitally supported appointment booking system can already be used, as well as automated dialog guidance on the care request [3]. While digitally linked medical history forms are used in the waiting room, decision support systems help with diagnosis and telemedicine applications with treatment. Patients can be issued an e-prescription, leading to fewer visits being required. Administrative processes of a general practice such as materials management or duty scheduling can also be digitally supported. The use of digital solutions can contribute to high-quality healthcare, for example, by improving patient safety [1]. It also relieves the burden of routine tasks in the general practice, so that more intensive attention can be paid to patient care [12]. Despite the potential of digitalization, implementation within a general practice is associated with challenges [13]. The authors Cresswell and Skeih [14] identified socio-cultural, organizational, and technological factors as obstacles to successful digitalization. The lack of digital skills among physicians is just one example of a multitude of barriers [13]. Consequently, there are many levers for promoting digitalization. This can pose challenges for decision makers in healthcare.

### 1.2. Maturity Measurements as a Roadmap for Digital Transformation 

Providing all stakeholders involved in the digitalization of primary care with a path to successful implementation would offer orientation in the transformation process. Maturity models have established themselves as strategic elements for organizations, showing them the direction along a development path [15]. They have been developed “to assess the maturity (i.e., competency, capability, level of sophistication) of a selected domain based on a more or less comprehensive set of criteria” [15] (p. 1). Maturity models are state descriptions with the goal of achieving maturity, i.e., “being complete, perfect, or ready" [16] (p. 83). They are usually based on a sequence of stages or phases that form the path from the initial state to maturity [17,18]. Maturity models originated in software development. The best-known maturity model is the Capability Maturity Model (CMM), which was developed in 1986 by the Software Engineering Institute (SEI) of Carnegie Mellon University in Pittsburgh and describes five maturity levels of software development processes [19]. At the first level, processes are unpredictable, have little control, and are reactive. At the fifth level, the organization is dedicated to the continuous search for process improvement [19]. The CMM formed the basis for many other maturity models in the past, e.g., in areas such as business process management [20] or knowledge management [21]. Healthcare institutions also use maturity models, including for benchmarking, self-assessment, change management, or organizational learning [22,23]. The World Health Assembly further points to the benefit of maturity assessments to identify areas for improvement. They therefore call on member states to evaluate the use of digital technologies. [24].

Several maturity models have recently been developed for the area of inpatient care, especially hospitals. The most widely used maturity model internationally in this context is the Electronic Medical Record Adoption Model (EMRAM) of the Healthcare Information and Management Systems Society (HIMMS). HIMMS describes it as a “strategic roadmap for [...] digital transformation journey toward optimizing organizational performance outcomes” [25] (p. 1). With the help of eight stages, it maps the path to a paperless hospital using electronic medical records [26]. National health institutions have also already taken up the assessment of hospital digitalization to improve decision-making for funding measures based on it. For example, the German Federal Ministry of Health has commissioned the “DigitalRadar Krankenhaus” consortium to evaluate the state of digitalization [27]. Another example is the Digital Maturity Self-Assessment of the NHS in Great Britain [28]. In parallel with the development of maturity models, academia continues to explore the notion of digital maturity in hospitals and how it is made measurable. In a recent study, Duncan et al. [29] examined the dimensions used in maturity models to measure digital maturity and have summarized them in a new framework. 

### 1.3. Objective

The large number of articles published for the area of digital maturity of hospitals underscores the progress of science in the inpatient sector. We take this as an opportunity to provide an overview of the state of research on digital maturity in primary care. GPs should be given equal consideration in terms of their level of digitization due to their position in the healthcare system. We want to understand how a maturity measurement can look as a roadmap for GPs in digital transformation. To this end, we examine how digital maturity of GPs has been described and measured in studies, how maturity models are applied, and whether there is evidence of correlations between digital maturity and outcomes within primary care. The aim of this work is to lay a foundation for further work on the digital maturity of GPs. 

## 2. Materials and Methods

To examine the state of research to date, we used a scoping review according to Arksey and O’Malley [30], considering the reporting scheme for PRISMA-ScR [31]. A scoping review is a type of review with the aim of obtaining an overview of the state of the literature on a research topic at the outset. It is primarily used to investigate a topic area that tends to be exploratory and rather broadly formulated and to derive research needs [32]. In contrast to systematic reviews, the focus is less on detailed questions about a topic area that has already been researched further and on the evidence within the sources. Thus, the requirements for literature selection are also lower; gray literature can also be included in the analysis. Grey literature, in the context of this publication, are white papers, reports, and theses without reference to the major publishers and journals (Directory of Open Access Journals), without impact factor, and without commercial reference, for example, to industrial companies. Arksey and O’Malley [30] identify five core steps for a scoping review: identifying the research question, identifying the relevant studies, selecting the studies, preparing the data, and reporting the results. Based on these steps, the methodological approach is described. 

### 2.1. Defining the Research Question

The identification of the research question was guided by the objective of our study. Primarily, our goal with the scoping review was to provide an overview of the state of the research literature on digital maturity in primary care, specifically among GPs. In particular, we aimed to shed light on the authors’ understanding of digital maturity and how it is operationalized. Therefore, we formulated our research question as follows: What does digital maturity mean for GPs in the context of digital transformation; how is it measured? On this basis, we secondarily looked at the measured results on digital maturity among GPs and whether there are signs of evidence of postulated benefits of a digital general practice in the literature.

### 2.2. Identifying Relevant Studies 

**Figure 1:**
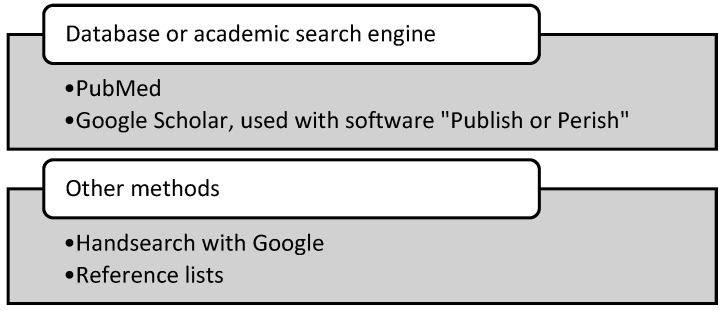
Search strategies.

After defining the research question, we identified the studies relevant to answering it. According to Arksey and O’Malley [30], it is important to find comprehensive (also unpublished) primary studies on the research field. In doing so, it is advisable to follow different search strategies. Following this approach, we conducted a literature search in September and October 2022 using different search strategies (Figure 1).

First, we searched the PubMed literature database, which contains a large proportion of all internationally published studies in the health and medical sector. However, the number of hits was very low, with fewer than 50 studies per search performed. For this reason, we expanded the search using the Google Scholar search engine. Google Scholar is a search engine specifically for academic literature and displays unpublished papers. The disadvantage of a search with Google Scholar, however, is the very high, often unmanageable number of hits, that contain a lot of gray literature. Therefore, the number of hits was limited to 100 results in this study using the software “Publish or Perish” [33]. The software primarily shows the most cited work. We then continued the literature search by searching the reference lists of the sources we had already found. Finally, to include more gray literature, we completed the literature search by a loose hand search using the Google search engine.

For the definition of search terms, the challenge was the different terminology used internationally to describe GPs. We therefore oriented ourselves to the European Health Information Portal [34]. An initial limited search in PudMed was undertaken to identify articles on the topic. The words contained in the titles and abstracts of the relevant articles and the index terms used to describe the articles were used to develop a complete search strategy for PubMed and Google Scholar. Guidance was likewise provided by the already more widely researched topic area of digital maturity among hospitals. As a result, we used the following Boolean search terms for our literature search using PubMed: “digital maturity” AND “general practice”; “digital maturity” AND “general practitioners”; “digital maturity” AND “family practice”; “digital maturity” AND “family practitioners”; “digital maturity” AND “primary care.” Instead of the term “digital”, the term “ehealth” was used analogously; the search continued with, for example, “ehealth maturity” AND “general practice”. In Google Scholar, we additionally added the term “health” to the search terms to show us search results from the health sector. The authors considered the inclusion of Scopus but decided against it due to an already achieved saturation of content by PubMed, as well as Google Scholar.

### 2.3. Selection of Studies 

For the literature selection, we defined inclusion and exclusion criteria in advance. To answer our research question, we only included papers that reported on digital maturity and its measurement among GPs. Furthermore, included works had to show a development plan towards a higher level of digitalization. In this way, we wanted to avoid the inclusion of papers that, for example, only reported on an adoption rate of specific technologies by GPs. Thus, for inclusion, the studies had to show differentiated levels of digitalization, e.g., by using a tiered maturity model or scale. In summary, therefore, we only included studies that provided information about what digital maturity is among GPs and how it is measured. That allowed GPs to be classified in terms of their level of digitalization. Formally, we excluded only papers that were not available in German or English, as well as reviews. We did not make any restrictions about the type of organization in which GPs work. Table 1 shows an overview of the inclusion and exclusion criteria.

**Figure 2:**
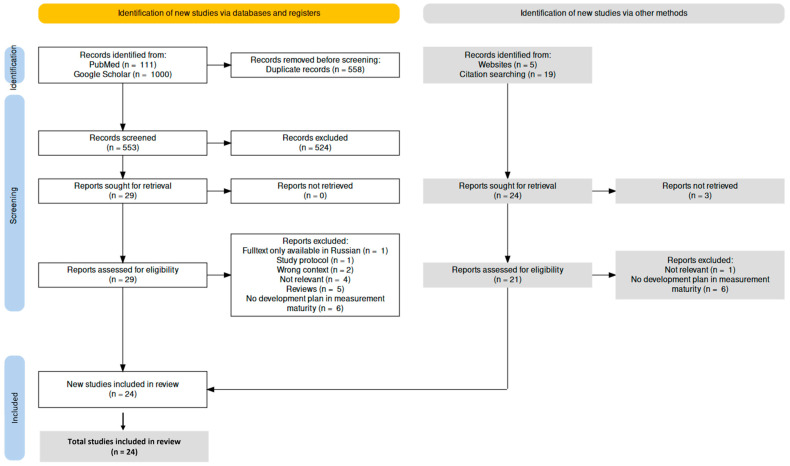
Preferred Reporting Items for Systematic Reviews and Meta-Analyses (PRISMA) flow diagram for articles identified, screened, and included in the review, made using the R ShinyApp.

The retrieved papers were first examined independently by both authors based on their titles and abstracts and using the inclusion and exclusion criteria. The full texts were then examined. If the full texts were not available, attempts were made to request them from the authors via ResearchGate. Any disagreements between the authors at any stage of the selection process were resolved through discussion. The study selection process is shown in a PRISMA flow diagram (Figure 2). The diagram was created using a free-to-use, open-source R package, and web-based Shiny app [35] and is based on recommendations from Rethlefsen and Page [36]. We have shown the results for the hand search with the Google search engine under “Websites”. 

### 2.4. Preparation of the Data 

Following the recommendations of Arksey and O’Malley [30], we next mapped the most important information about the identified papers. Using the database program Excel, we first summarized the papers using general information such as author, publication date and location, and study type. Second, we analyzed the papers with respect to our research questions to be answered. We began by examining the general context of digital maturity described in the studies. We then addressed the operationalization of maturity and the development of maturity levels in the measurement models used. Lastly, we also looked at the results of measured digital maturity among GPs and the data collection methodology required for this.

### 2.5. Reporting the Results 

Reporting is critical for a scoping review to provide readers with an understandable overview of the state of the research literature to date [30]. We reported on the analyzed content described in Section 2.4 using a combination of numerical and thematic reporting. Where appropriate, we have presented the results in a summarized and visual form using tables and diagrams.

## 3. Results

The first part of our literature search, which we performed using PubMed and Google Scholar, yielded 1111 search results. After excluding duplicates, this number was reduced to 533, which was again reduced to 29 papers after screening the abstracts. A total of 10 of these papers met our requirements according to our predefined inclusion criteria after analysis of the full texts. In the second part, we were able to identify 5 papers through websites found using Google and 19 more through reference lists. After 3 papers could not be found as a full text and further papers were excluded, we included 14 additional papers in our study pool. In total, we included 24 papers in our scoping review. 

### 3.1. Study Characteristics 

**Figure 3:**
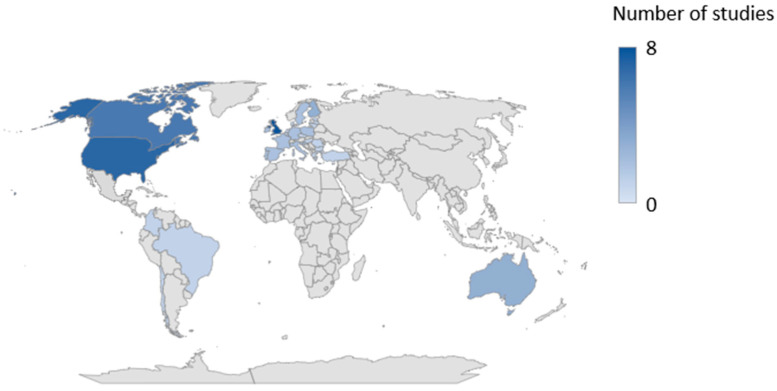
Geographical distribution of digital maturity examined in the included studies.

The included studies were published in the period 2000–2022, with most papers (n = 5) published in 2022. In terms of study location, the majority of included studies were from the Anglo-American-speaking world. These included the United States (n = 6) and the United Kingdom (n = 6), followed by Canada (n = 5). Two papers examined the digital maturity of GPs in different countries. This included, for example, a benchmarking exercise commissioned by the European Commission on the use of eHealth by GPs in 27 EU member states. Figure 3 shows the countries from which the studies originated or in which digital maturity among GPs was examined.

Finally, nine of our included papers were gray literature. These included unpublished papers from initiatives by governmental institutions (n = 4) and dissertations (n = 2). 

### 3.2. Context of Digital Maturity

**Figure 4:**
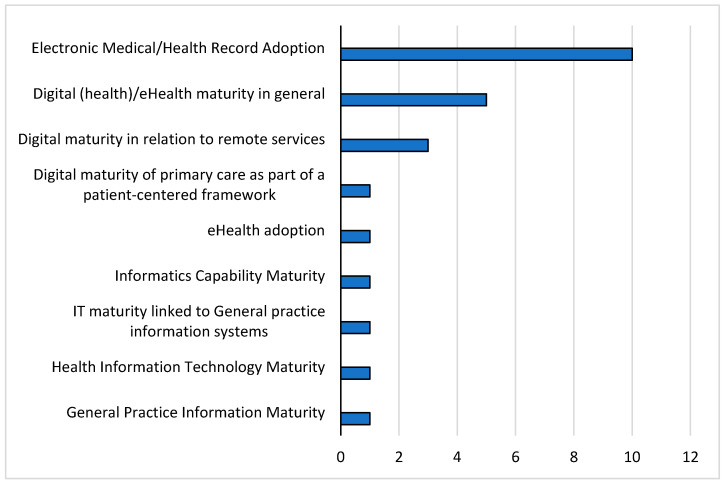
Context of digital maturity described by context categories.

In the included papers, the digital maturity of GPs was understood and examined differently. As a result of our review, digital maturity could be categorized into nine context categories (Figure 4). 

In most of the included studies (n = 10), the measurement of digitalization was equated with the adoption and use of electronic medical records (EMR) and electronic health records (EHR), respectively. These included the HIMSS Outpatient Electronic Medical Record Adoption Model (O-EMRAM), an adaptation of the classic EMRAM for ambulatory healthcare settings [37]. Other authors investigated the level of maturity in electronic medical records using a maturity model adapted from EMRAM [38,39,40,41]. In further studies, physicians or general practices were classified into different user or usage categories [42,43,44,45]. Five studies explicitly used the term digital maturity or ehealth maturity and examined these without a separate focus on a single technology for GPs [46,47,48,49,50]. These resulted primarily from national initiatives or government efforts to measure the progress of digitalization in ambulatory care. These included, for example, an NHS England maturity measurement [48] or Victoria’s Digital Health Maturity Model [46]. The latter stemmed from efforts by the Australian Victorian Department of Health to measure the digital maturity of all healthcare facilities, not just hospitals. Three studies considered digital maturity in relation to the use of remote services [51,52,53]. Flott et al. [54] developed a framework for measuring digital maturity across the patient care pathway and described measurement for primary care in that context. How well healthcare organizations collect, manage, and share information has been examined with the Informatics Capability Maturity, described by Liaw et al. [55]. Finally, individual studies addressed the adoption of ehealth applications in general [5], the maturity of health information technology (HIT) in small- and medium-sized physician practices [56], IT maturity in connection with physician information systems [57], and the General Practice Information Maturity Model (GPIMM) for the information management of a general practice [58]. 

### 3.3. Operationalization of Digital Maturity 

Within the different context categories, the method in which digital maturity was operationalized differed (see Appendix A Table A1, third column). Overall, across all included papers, digital maturity was associated with technology in a general practice. This meant, for example, measuring whether technologies were present or whether—and to what extent—and with what functions they were used. For example, in the Chong et al. [38] model, GPs were asked about the maturity of their appointment scheduling system. Other technical aspects included system interoperability [50,54], system stability [56], or privacy and security [46,49]. The technological focus of maturity was represented to varying degrees in the topics surveyed. In particular, in maturity measures for EMR such as the O-EMRAM, maturity was almost exclusively associated with the presence and use of different functions of the application [37]. Hermanns [57], in turn, has linked the technical capabilities of a physician information system to overall IT maturity in his work. However, even in studies without a focus on an application, the focus on technology use was found. In the benchmarking for the European Commission, the adoption of information and communication technologies (ICTs) was measured [5]. Teixeira et al. [50], who wanted to measure the digital maturity of GPs as a whole, also used usage as an indicator. Away from the technological focus, the number of studies that explicitly used sociocultural and organizational factors to measure digital maturity was smaller. Individual competencies of physicians and practice staff [49,50,54], attitudes toward digitalization and expected positive outcomes [47,50], or topic areas such as governance, management, and strategy alignment [46,52,55] were measured. 

### 3.4. Maturity Development 

Maturity models or categorizations were used in 19 studies to represent maturity development, with each level described separately (see Appendix A Table A1, fourth column). Other studies used scales without further description (n = 4). Maturity development, with or without further description of stages, both commonly assume a universally valid developmental path. This means that for all characteristics to be measured, the maximum maturity level to be reached, as well as the path to reach it remains the same. The model for measuring the maturity of physician information systems occupied a special position. Instead of a generally valid categorization for all indicators to be measured, specific maturity levels were defined for each focus area [57]. Where individual maturity categories were described in the studies, the range of levels used was from three to eight. The O-EMRAM had the most differentiation with eight levels. Five or six levels were most frequently used (n = 13). Further, the maturity levels used in the studies differed by context and operationalization of digital maturity. In the studies that looked at electronic health records, the lowest level generally meant a paper-based general practice, while full maturity meant full use of the system [37,38,39,40,41]. This was similarly found in the GPIMM by Gillies et al [58]. In studies that focused on technology, the ability to share information externally with organizations in other care sectors was associated with a high level of maturity. Where digital maturity was associated with organizational culture and leadership, the lowest maturity was expressed by, for example, a traditional general practice with limited leadership or vision [51,52,53]. In two other studies, the highest level of maturity was attributed to coordinated, planned initiatives, and a continuous improvement process within the organization [46,55]. Last, Wallace used Maslow’s pyramid of needs to rank the maturity level of HIT in physician practices. The top level of maturity, which in Maslow’s model is human self-actualization, was expressed by a paradigm shift in technology in Wallace’s model. That is, HIT permanently changes the organization and the way of working in the general practice [56].

### 3.5. Maturity Survey and Results 

Most studies proposed quantitative methods for measuring digital maturity (n = 15). These usually included standardized surveys with questionnaires. Twenty included papers measured the maturity of GPs or general practices, which varied by context (see Appendix A Table A2; third column). In a study by the European Commission, 5793 GPs from 27 participating member states participated in a survey on information and communication technology use and had an average score of 2.131 out of 4 [5]. In the paper by Teixeira et al. [50], who analyzed survey data from 1600 GPs from 20 countries, the median digital maturity score was 4 out of 6. 

In the included papers, a direct relationship between digital maturity and outcomes within primary care was sporadically examined. In the classification of electronic health record user types described by Miller et al. [42], “System Changer” benefited from improved quality of care, financial benefits, and time savings. They advocated for process changes related to use, thus, representing the highest level in the model. Wallace [56] pointed to benefits of mature IT. These ranged from user satisfaction with stability of systems to time savings, lower costs, and faster transfer of information, provided the systems allow for cross-organizational integration. A study by Greenhalgh et al. [52] showed that a high level of digital maturity in general practices does not necessarily have a positive effect on patient care. For example, practice staff in a digital medical practice were concerned about lack of access by less digitally savvy patients and reported cases in which patients had to visit an emergency room due to lack of access. 

## 4. Discussion

Maturity measurements enable a roadmap for the digitalization of GPs. While the focus of maturity measurements to date has been on the inpatient sector, this study deliberately looks at outpatient care. We addressed the research question of how digital maturity among GPs is understood and measured in previous research. Our results can be interpreted as follows: 

First, the research literature on digital maturity in this area is still in its infancy. We found only one scientifically published study describing the measurement of digitalization of GPs as a whole [49]. In addition, this study was published in August 2022 and was therefore brand new at the time of the literature search. However, the aim of the above study was to report on the national development of eHealth maturity. Although indicators and their derivation were presented, there is a lack of a published study that explicitly explores the dimensions of digital maturity among GPs and their operationalization. This is in contrast to digital maturity among hospitals, where research is much more advanced and maturity model frameworks exist, as in the study by Duncan et al. [29]. Thus, our results highlight a research gap. It is noted that research on digital maturity of GPs lags that of hospitals. This could be due to the higher healthcare expenditure in inpatient care compared to outpatient care [59]. Healthcare provision in hospitals substantially differs from providing conventional services, as they are like an ecosystem within the health ecosystem [60]. The evaluation of digital maturity in this closed system has advantages over the highly heterogeneous system of outpatient treatment. Depending on the type of health system, GPs are not employees, but rather, micro-entrepreneurs; they run their own medical practice. Business, legal, and technical decisions have to be made alone [61]. In contrast, hospitals usually have a middle management that can devote itself, for example, to digitalization issues [62].

Research on digital maturity in the context of expected process improvements and healthcare improvement seems to have been more valuable in the past. With the guiding principle of “outpatient before inpatient,” countries with a strong focus on inpatient care, such as Germany, want to strengthen outpatient care [63]. It is therefore understandable that the digital maturity of GPs is also becoming the focus of research. Our findings confirm the interest in digital maturity of GPs. There were several unpublished papers, starting from national initiatives, that have attempted to measure digital maturity in primary care. Future research should build on the growing interest and examine digital maturity of GPs.

Following on from this, the results showed that, despite initial attempts, there is still no uniform understanding of the digital maturity of GPs. The context of the maturity studied varied widely. Maturity was examined in general, as well as more specifically, by the maturity of technologies such as the physician information system or the electronic health record. The latter took center stage in our review. Looking at the publication data, it was noticeable that more recent work was devoted to a holistic understanding of digital maturity, while older work focused primarily on technologies such as electronic health records. We attribute this change to a shift in the fundamental understanding of digitalization. Digitalization—or in this case digitization—stems from a technical understanding that was described by the transformation of analog into digital processes [64]. The use of the term digital transformation expresses the profound change with the impact of digitalization on people and the organization. Digital transformation not only encompasses the introduction of IT technologies. It is therefore not surprising that the operationalization of digital maturity is also undergoing a change. Future research should address operationalizing digital maturity of GPs. By exploring and testing dimensions to measure maturity, a valuable contribution could be made to the development of a consistent digital maturity model.

In connection with the section above, our findings showed parallels to the level of digital maturity in inpatient care. We found that, so far, primarily the use and capabilities of technologies have been equated with maturity. Similar findings can be seen in maturity models of hospitals. Representative examples include the EMRAM of the HIMSS or the Digital Maturity Self-Assessment of NHS England [26,28]. Although models such as the EMRAM are widely used internationally, they have limitations. Criticisms include equating digital maturity with digital infrastructure. Human or organizational capabilities would be neglected [65]. This can be explained by the lack of a holistic understanding of digital maturity, as noted under second. Most of the included papers focused exclusively on electronic health record technology. Moreover, several of the papers we considered were based on the EMRAM of the HIMSS. It is therefore not surprising that we found similar limitations to maturity models in inpatient care.

Furthermore, we would like to discuss the last part of our research question, which asks about signs of evidence of postulated benefits of a digital general practice. This question requires researchers to ask whether there are already existing effect measures for digital health in general. There is a lot of controversial discussion, e.g., to use health technology assessment for digital health [66,67]. One would also have to take the use of Patient-Reported Outcome Measures (PROMs) and Patient-Reported Experience Measures (PREMs) into account [68]. New frameworks like NICE (National Institute for Health and Care Excellence) in the UK try to bring evidence to the digital health world [69]. With regard to our results, no attempts could be identified in the maturity approaches to demonstrate efficiency and effectiveness improvements.

Finally, our scoping review showed that there is a need for research on the expected relationship between digital maturity and impact on primary care. Overall, few studies have addressed the question of whether high levels of digital maturity are associated with positive patient care outcomes. Consequently, there is a lack of studies on the evidence of a digitally advanced general practice. Evidence of improvements in care is important above all, as it motivates all relevant stakeholders to increase their digitalization efforts. For the future, it will be important not only to understand digital maturity and operationalization, but also to study the impact on care.

### Limitations 

The conclusions are to be classified against the background of limitations of our scoping review. It is possible that, despite our broad search strategy and the use of search engines such as Google Scholar, we did not find all literature relevant to the topic area. Similarly, the selection of search terms used and the inclusion and exclusion criteria may have led to limitations. Flott et al. [54] noted that the term digital maturity is often defined in the literature in terms of individual digital technologies rather than as a whole. For our literature review, this would have meant either an immense expansion of search terms or a predefinition of specific terms related to selected digital technologies. Since this would have led either to problems in the evaluation due to too many studies or to misinterpretations due to too strong a weighting on individual technologies, we decided against it. The fact that we came across studies with a technological focus despite general terms confirms our approach. In addition, we included a range of gray literature for our scoping review. Although unpublished papers can in principle be included, their weight in the context of the evaluation is lower. However, they underline the exploratory, largely unexplored research field that we have demonstrated in a transparent and reproducible procedure.

## 5. Conclusions

This review provides an overview of the state of research on digital maturity in primary care and, specifically, among GPs. Our findings revealed that research on digital maturity is nascent. Research gaps exist regarding a unified understanding of digital maturity, its operationalization and measurement, and its impact on primary care outcomes. 

Future research should aim to develop a consistent and validated model for measuring the digital maturity of GPs. This requires a deeper understanding of the meaning of GPs’ digital maturity. Future work should therefore focus on the dimensions of digital maturity. To this end, for example, surveys could be conducted among relevant primary care stakeholders on the one hand. On the other hand, dimensions of digital maturity from the inpatient sector should be examined for transferability. Comparisons should therefore be made between the digital maturity of a hospital and a general practice. In particular, the human factor, which has been little represented to date, but is considered essential, should be given attention for future models. Finally, the relationship between digital maturity and positive effects on patient care needs to be investigated more intensively. Until effectiveness is proven, digitalization efforts will have little success. Scientific evidence of improved patient care and financial benefits, on the other hand, can give physicians incentives to embrace digitalization. They also legitimize policymakers to allocate more resources to digitalization in primary care to improve healthcare in the long term. 

## Figures and Tables

**Table 1:** Inclusion and exclusion criteria.

Inclusion Criteria	Exclusion Criteria
Publication in German or English	Publication in languages other than German or English
Addressing digital maturity and its measurement among general practitioners	Examining digital maturity among other physician groups (e.g., specialists) or in other health care sectors (e.g., inpatient care)
Maturity measurements enable GPs to be ranked in terms of their level of digitalization	Loose measurement of the level of digitalization without using a development plan
	Reviews

## Data Availability

According to the Guideline for Good Scientific Practice and Research Data Management of Witten/Herdecke University, data can be requested by contacting the corresponding author.

## Appendix A

**Table A1:** Overview of the maturity measurements used.

Maturity Context	Maturity Model	Operationalization	Maturity Development	Refer-ence
EMR/HER Adoption	Outpatient-EMRAM (O-EMRAM)	Used EMR capabilities	8 levels from 0–7 - Level 0 = Paper Chart-Based - Level 7 = Complete EMR: External Health Information Exchange, Data Analytics, Governance, Disaster Recovery	[37]
	EMR Maturity Model	Used EMR capabilities	6 levels from 0–5 - Level 0 = Processes are primarily paper-based. - Level 5 = Use of portals, hubs, attachment to provincial e-health platforms sharing data from the EMR	[38]
	EMR Adoption Model	Used EMR capabilities	6 levels from 0–5 and 1–6, respectively (Tagg recoded the original scale) - Level 0: Traditional paper-based practice. Charts on paper, results received on paper. - Level 5: Full EMR that is interconnected with regional/community Hospitals, other practices, labs, and pharmacists for collaborative care	[39,40,41]
	Clinical Value Model	Used EMR-Capabilities	5 levels from 1–5 - Level 1: Front Office Administration: Basic billing and scheduling system in use—little or no clinical data or point-of-care us - Level 5: Community Shared Care: Data transfer enables effective shared care between GP and specialists and other care providers	[70]
	Miller et al. Model	Used EMR/EHR-Capabilties	5 EMR/EHR user types of physicians: - Type 1: Viewer: Physicians who only use the EHR’s display function - Type 5: System Changer: Physicians who advocate for workflow changes through the EHR	[42,43]
	Lanham et al. Model	- Degree of use of EHR functions - Degree of EHR-supported communication with others - Frequency with which EHR use changed within the team (i.e., change in use due to new EHR system features)	3 EHR use categories at the practice level and physician level from low to high	[44,45]
Digital (health)/eHealth maturity in general	eHealth Maturity scale by Haverinen et al.	3 areas, 16 indicators - Applications area: e.g., electronic patient record - Area of regional integration: e.g., exchange of clinical care information - Area of data security and ICT skills: e. g., e-ID and signature	Each indicator is measured on a scale from 0 up to 10	[49]
	Digital maturity framework adapted from Flott et al. [54]	- Utilization - Resources and ability (organizational) - Resources and ability (individual) - Interoperability - General assessment methodology - Impact	Maturity scale from 0–6	[50]
	Digital health maturity assessment for general practice by Gippsland Primary Health Network	- Infrastructure - Capabilities - Readiness - Willingness	3 levels from 1–3 - Level 1: Foundational: focus on the availability of basic digital health infrastructure - Level 3: Advanced: focus on the implementation of new digitally-enabled models of care for remote monitoring of patients with chronic disease, social prescribing and mental health	[47]
	Victoria’s Digital Health Maturity Model	The model is built around nine pillars. “Governance and stewardship” sits across the nine pillars: - Organizational capability - IT operations and infrastructure - Level of digitization and functional adoption - Security and privacy - Information sharing and integration - Data and analytics - Consumer participatory health - User experience - Innovation	5 Levels from 1–5 - Level 1: Initial: Digital health outcomes are unpredictable, reactive, and poorly controlled. - Level 5: Transformative: Digital health outcomes are realized through coordinated and planned initiatives that form part of a continuous improvement loop.	[46]
	NHS‘ Digital Primary Care Maturity Assurance Model	IT capabilities	3 categories: - Category 1: Basic and Prescribed IT: technologies and systems required for the delivery of basic primary care services. - Category 3: Changing primary care: including technologies and systems that enable new models of care, cross-organizational collaboration	[48]
Digital maturity in relation to remote services	Digital maturity scale for healthcare organizations in relation to remote services	- Infrastructure - Capability - Readiness	5 Levels from 1–5 - Level 1 Traditional (reactive): including limited leadership or vision, low use of video and telemedicine, lack of key infrastructure. - Level 5: System-oriented (expansion and proliferation): including strong and beyond organization strategy and vision for remote services, high digital capabilities, employee participation in development and evaluation of remote services	[51,52,53]
Digital maturity of primary care as a part of a patient-centered framework	Patient-Centered Framework for Evaluating Digital Maturity	- General evaluation methodology - Resources and ability - Usage - Interoperability - Impact	Point scale from 0–14	[54]
eHealth adoption	Composite index for overall ICT adoption	Used ICT-Capabilities - EHR - Health Information Exchange - Telehealth - Personal Health Record	Composite eHealth index for ICT adoption. 0 = Not known 4 = Routine use	[5]
Informatics Capabilty Maturity	Informatics Capability Maturity Model	- Data collection, integration and management in HIS/EHR - Information sharing in the health neighborhood - Managing health information and Communication technology implementation and change - Data Quality Management and Information Governance - Using health “business intelligence” to improve care and population health	5 levels from 1–5 per indicator - Level 1: Systems and processes are not fully reliable or coordinated - Level 5: Facilitates innovation with enterprise level engagement.	[55]
IT maturity linked to General practice information systems	Hermanns’s Focus area maturity model to assess IT maturity in general practices	Focus areas and capabilities of a physician information system	Each focus area has its own number of specific maturity levels, and the overall maturity level of a physician information system is expressed as a combination of the maturity levels of these focus areas	[57]
Health Information Technology Maturity	Wallace’s adapted SMPP IT Value Hierarchy Model	Including HIT applications used, integration of HIT applications, system stability, quality and speed of information reception, attitude toward the system, interaction with the system, patient interaction	5 levels, depending on the distinctiveness of the IT - Stage 1: Infrastructure and connectivity needs - reactive IT, basic infrastructure with no standards and little to no IT policies - Stage 5: Paradigm shift - IT changes the organization and the way it works	[56]
General Practice Information Maturity	General practice information maturity model	- Use of computers - Use by staff - Extent and quality of coding - System usage (impact of the system on the practice’s working methods). - Implementation of the EMR	6 levels from 0–5 - Level 0: Paper-based - The practice has no computer system - Level 5: The practice is completely paperless, except where paper records are a legal requirement.	[58]

**Table A2:** Results of maturity measurement.

Maturity Context	Maturity Model	Maturity Results	Reference
EMR/EHR Adoption	Outpatient-EMRAM (O-EMRAM)	Not available (N/A)	[37]
	EMR Maturity Model	More than half were at level 1 or 2 for current EMR maturity level.	[38]
	EMR Adoption Model	Overall EMR adoption - Source 39: 2.3–3 - Source 40: 2.17 and 2.87, respectively - Source 41: 4.01	[39,40,41]
	Clinical Value Model	2.5 and 3.5, respectively	[70]
	Miller et al. Model	- Source 42: Most physicians were Arrivers (Type 4) - Source 43: Most physicians were Viewers (Type 1) or basic users (Type 2)	[42,43]
	Lanham et al. Model	- Source 44: Tends to be medium EHR use in general practices - Source 45: Two-thirds physicians categorized as medium EHR users.	[44,45]
Digital (health) /eHealth maturity in general	eHealth Maturity scale by Haverinen et al.	- Predominantly high level of maturity per indicator (8–10) - Lowest maturity level = intensity of use of electronic appointment booking (1 out of 10)	[49]
	Adapted digital maturity framework developed by Flott et al. [54]	Median digital maturity level of 4	[50]
	Digital health maturity assessment for general practice by Gippsland Primary Health Network	Just under two-thirds of the practices surveyed achieved Level 2.	[47]
	Victoria’s Digital Health Maturity Model	N/A	[46]
	NHS‘ Digital Primary Care Maturity Assurance Model	N/A	[48]
Digital maturity in relation to remote services	Digital maturity scale for healthcare organizations in relation to remote services	- Source 51: different; no further details - Source 52: varied maturity levels. Most general practices had maturity level 4 - Source 53: different; no further details	[51,52,53]
Digital maturity of primary care as a part of a patient-centered framework	Patient-Centered Framework for Evaluating Digital Maturity	N/A	[54]
eHealth adoption	Composite index for overall ICT adoption	EU average of the composite index is 2.131	[5]
Informatics Capabilty Maturity	Informatics Capability Maturity Model	- Data collection, integration, and management in HIS/EHR had the highest overall maturity (level 3 or 4). - Using health “business intelligence” to improve care and population health showed the lowest maturity (1–3).	[55]
IT maturity linked to General practice information systems	Hermanns’s Focus area maturity model to assess IT maturity in general practices	The maturity of physician information systems from individual manufacturers was measured. These showed different levels of maturity.	[57]
Health Information Technology Maturity	Wallace’s adapted SMPP IT Value Hierarchy Model	Most practices met the requirements of level 3.	[56]
General Practice Information Maturity	General practice information maturity model	Most practices had a maturity score of 1.	[58]
